# Nutritional Intake Influences Zinc Levels in Preterm Newborns: An Observational Study

**DOI:** 10.3390/nu12020529

**Published:** 2020-02-19

**Authors:** Gianluca Terrin, Giovanni Boscarino, Maria Di Chiara, Silvia Iacobelli, Francesca Faccioli, Carla Greco, Elisa Onestà, Giulia Sabatini, Andrea Pietravalle, Salvatore Oliva, Maria Giulia Conti, Fabio Natale, Mario De Curtis

**Affiliations:** 1 Department of Maternal and Child Health, University of Roma La Sapienza, 00161 Rome, Italy; giovanni.boscarino@yahoo.com (G.B.); maria.dichiara@uniroma1.it (M.D.C.); francesca.faccioli@hotmail.it (F.F.); g.carla80@gmail.com (C.G.); elisa.onesta@gmail.com (E.O.); sabatinigiulia0@gmail.com (G.S.); apietravalle@gmail.com (A.P.); salvatore.oliva@uniroma1.it (S.O.); mariagiulia.conti@uniroma1.it (M.G.C.); fab.natale@libero.it (F.N.); mario.decurtis@uniroma1.it (M.D.C.); 2 Service de Néonatologie. Centre Hospitalier Universitaire Sud Réunion, BP 350, 97448 Saint-Pierre Cedex, La Réunion, France; silvia.iacobelli@chu-reunion.fr; 3 Centre d’Etudes Périnatales Océan Indien (CEPOI). Centre Hospitalier Universitaire Sud Réunion, BP 350, 97448 Saint-Pierre Cedex, La Réunion, France

**Keywords:** parenteral nutrition, energy intake, protein intake, refeeding syndrome, VLBW

## Abstract

(1) Background: Zinc is a key element for protein synthesis in preterm newborns. Early aggressive nutrition, promoting protein synthesis, may increase zinc consumption; (2) Methods: We performed a prospective observational study, to assess the relationship between early macronutrients intake and serum zinc levels, in preterm newborns with Gestational Age (GA) of 24–35 weeks, consecutively observed in Neonatal Intensive Care Unit (NICU). (3) Results: We enrolled 130 newborns (GA 31.5 ± 2.8). A significant negative correlation between serum zinc level at 28 days of life and energy (r −0.587, *p* < 0.001) and protein intake (r −0.556, *p* < 0.001) in the first week of life was observed. Linear regression analysis showed that zinc levels depended on energy (β −0.650; *p* < 0.001) and protein (β −0.669; *p* < 0.001) intake given through parenteral nutrition (PN) in the first week of life; (4) Conclusions: zinc status of preterm neonates was influenced by early protein and energy intake. An additional zinc supplementation should be considered when high protein and energy intake are received by preterm newborns in the first week of life.

## 1. Introduction

Zinc is one of the most abundant trace elements in humans [1]. The particularity of this nutrient is, certainly, the even distribution throughout the body as a key component of enzymes, transcription factors, hormonal receptor sites and biologic membranes [2,3]. Functionally, zinc participates in gene expression, neurotransmission, apoptosis, macronutrients metabolism and in inflammatory responses [3,4]. All these functions are crucial during anabolic conditions, when additional processes, beyond basal metabolism, are needed for adequate growth. During the first weeks of life, very low birth weight (VLBW) newborns are at high risk of malnutrition and, consequently, of growth restriction. In order to promote anabolism, the recent guidelines for PN, recommend early introduction of high energy and protein intake from the first day of life [5,6]. This approach is named “aggressive” nutrition. It has been recently observed that improved protein synthesis—induced by an aggressive PN—is associated with a reduction in serum phosphate and potassium levels, due to their increased utilization during anabolic states [7]. Considering the crucial role of zinc in protein synthesis, we hypothesized that aggressive nutrition may also influence zinc metabolism in VLBW newborns.

In this study, we aimed to investigate the relationship between early nutrition and zinc levels in preterm newborns.

## 2. Materials and Methods

### 2.1. Study Design and Population

This is a prospective observational study, including all neonates born between 24 and 35 weeks of postmenstrual age (PMA), consecutively admitted to the NICU of Policlinico Umberto I Hospital, La Sapienza-University of Rome, from 24 December 2014 to 15 May 2016. We excluded newborns with major congenital malformations, inborn errors of metabolism, congenital infections, hospital discharge or death within the first 72 h of life, as well as infants of mothers with immunologic disorders, gestational diabetes, infectious or chronic diseases, or with a history of drug or alcohol abuse.

### 2.2. Zinc Levels Measurement

Serum zinc levels were measured at birth, from cord blood, and at 28 days of life, from neonatal peripheral blood. Cord clamping was not delayed. Blood samples were collected in dedicated tubes containing lithium heparin (S-Monovette^®^ Lithium Heparin Gel for Trace Metal analysis), avoiding contamination with dust and excluding samples with hemolyzed blood. Two aliquots of 0.2 mL serum were obtained by centrifugation from each blood sample and stored at −20° until the analysis was performed. Serum zinc measurement was performed in duplicate by means of an atomic absorption spectrophotometry (AAS), with a wavelength of 575 nm. Serum zinc levels were expressed in µg/dL. Measurements of zinc concentration in biologic samples were performed by investigators who were blinded to the objectives of the study and the clinical conditions of newborns.

### 2.3. Data Collection

Investigators who were not involved in the NICU clinical practice prospectively recorded—for each patient enrolled in the study—prenatal, perinatal, and postnatal data using a structured data form, until discharge, death or transfer to another hospital. Data on daily cumulative parenteral and enteral proteins, fats, electrolytes and caloric intake were reported in a specific data form. Modalities of enteral nutrition administration and feeding tolerance were also recorded. Gestational age (GA), birth weight (BW), type of delivery, twin pregnancy, antenatal steroids administration, Apgar score at 1st and 5th minute after birth, Clinical Risk Index for Babies (CRIB) II score, base excess on cord blood analysis, body temperature within 1 h after birth, occurrence of IUGR, pregnancy-induced hypertension and other relevant obstetric information were collected. Data regarding resuscitation at birth, need and duration of mechanical ventilation, occurrence of respiratory distress syndrome, administration of surfactant and patent ductus arteriosus (PDA) and mortality, were prospectively recorded [8]. Diagnosis of necrotizing enterocolitis (NEC), bronchopulmonary dysplasia (BPD), intraventricular hemorrhage (IVH), periventricular leukomalacia (PVL), retinopathy of prematurity (ROP), early and late onset sepsis proven by positive cultures and anaemia of prematurity, were performed according to the standard criteria, by physicians unaware of the study design and aims [9,10,11,12,13]. Growth velocity during hospitalization and discharge were decided by the physicians, according to American Academy of Pediatrics criteria [14,15].

### 2.4. Nutritional Protocol

Parenteral Nutrition was started immediately after birth as a soon as a vascular access was obtained. Individualized formulations prepared in the hospital pharmacy were administered. Both the initial amount and the rate of amino acids (AA) and calories increase were decided daily by the prescribing physician in charge of the patients, according to a specific nutrition protocol available in NICU (Table S1).

All stable infants (defined by: normal SpO_2_, heart rate, arterial pressure, body temperature, absence of apnea in the previous four hours of life) received enteral feeding starting within the first 24–48 h of life (10 mL/Kg/day divided in 4–8 feeds) using a formula specific for preterm newborns routinely given in our NICE (Pre-Nidina Nestlè^®^: Proteins 2.9 g/dL, Lipids 4.0 g/dL, Energy 8.1 g/dL, Sodium 51 mg/dL, Potassium 119 mg/dL, Calcium 116 mg/dL, Phosphorus 77 mg/dL, Iron 1.8 mg/dL, Zinc 1.2 mg/dL) [16] or fresh, unfortified human milk of own mother was administered whenever available. Donor breast milk during the study period was not available. Human milk fortification was added once the infant was established on full enteral feeds of 120 mL/kg/day. Stomach residuals were measured prior to each feed. The cumulative gastric residual volume was calculated over a 24-h period. Stomach residuals without blood or bile were replaced. Enteral nutrition (EN) was withheld in case of abdominal wall erythema, absence of bowel sounds, blood in the stools, bile or blood in aspirates associated with radiological marker of NEC-Bell stage ≥ II [17,18]. In the absence of feeding intolerance, the EN was increased daily by 10–20 mL/kg.

Until full enteral feeding was reached, PN was administered through a central vascular access in all infants to ensure adequate intake of fluids, electrolytes and nutrients. Infants on PN received 350 µg/kg/day of zinc (Peditrace, Fresenius Kabi^®^, Bad Homburg, Germany), starting from the third day of life. We calculated that EN provides about 1.7 mg/Kg/day of zinc, when full enteral feeding (140 mL/Kg/day) is reached. Nutrient contents of parenteral solutions and formula were calculated based on the published manufacturer’s labels.

We calculated the percentile curves of both energy and protein intake in the first week of life. Thus, we considered any value equal or above 75th percentile, as high energy and high protein intake. In particular, we considered “high” 420 Kcal/Kg/week of energy and 15 g/Kg/week of proteins given by PN. When it comes to total intake, we considered 600 Kcal/Kg/week of energy and 18 g/Kg/week of proteins as the “high” total energy and protein intake.

### 2.5. Ethics

We conducted the study in conformity with the World Medical Association Declaration of Helsinki for medical research involving human subjects, and the Ethics Committee approved it with the number 91613. We collected anonymized data and a written informed consent was obtained from the parents of each enrolled infant.

### 2.6. Statistical Analysis

Statistical analysis was performed using Statistical Package for Social Science software for Microsoft Windows (SPSS Inc, Chicago, IL, USA), version 22.0. We checked for normality using a Shapiro-Wilk test. The mean and standard deviation or median and interquartile range summarized continuous variables. We used a chi-square test for categorical variable, *t*-test, Mann-Whitney and Wilcoxon test for paired and unpaired variables. After checking for assumptions, linear regression analysis with a stepwise method was used to study the possible influence of the different variable (BW, VLBW or not-VLBW, sex, IUGR, antenatal steroid, weight gain, caloric and protein intake in the first week of life) on serum zinc concentrations at 28 days of life. Correlation was assessed with categorical variables by Wilcoxon rank sum tests and with continuous variables by Pearson correlation. The level of significance for all statistical tests was 2-sides (*p* < 0.05).

## 3. Results

We considered 130 eligible newborns during the study period. Twenty-seven were excluded because of death or transfer within the first 72 h of life, major congenital malformations, inborn errors of metabolism, congenital infections or because they were born from mothers with immunologic disorders, gestational diabetes, infectious or chronic diseases or with a history of drug or alcohol abuse.

The main clinical characteristics of the enrolled newborns were summarized in Table 1.

Measurements of serum zinc concentration during the first 28 days of life were reported in Table 2. VLBW newborns showed lower serum zinc levels at 28 days of life when compared with newborns with birth weight ≥ 1500 g and VLBW subjects received more significant energy and protein intake by PN compared to the latter ones (Table 2). We observed a greater decrease in zinc concentration during the first 28 days of life in accordance with birth weight (Table 2). In comparison with non-VLBW newborns, we observed that VLBW subjects had an increased growth velocity.

**Figure 1:**
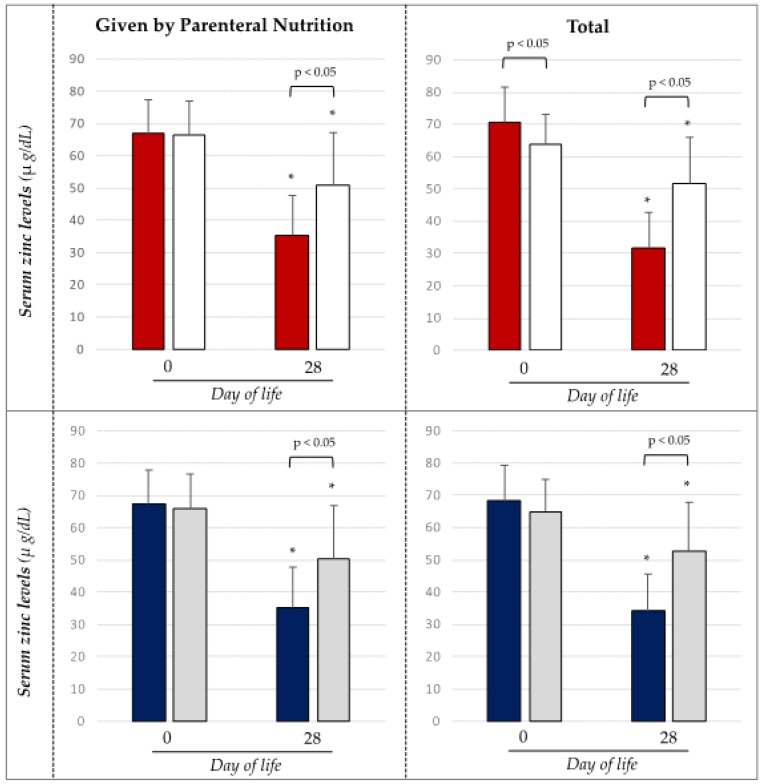
Level of serum zinc preterm newborns according to energy and proteins intake of the first week of life.

Newborns receiving high protein and energy intake during the first week of life showed significantly lower serum zinc levels at 28 days of life (Figure 1).

Interestingly, we observed a higher decrease in zinc concentration during the first 28 days of life according to energy and protein intake received early in the life (Table 3).

**Figure 2:**
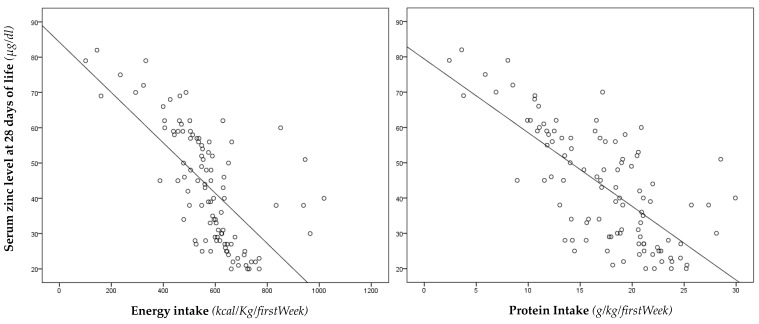
Correlation between serum zinc levels at 28 days of life with energy and protein intake.

We also observed a significant negative correlation between serum zinc levels at 28 days of life and total energy and protein intake, in the first week of life (Figure 2).

A negative correlation was observed between serum zinc levels at 28 days of life and parenteral energy (r −0.587, *p* < 0.001) and protein intake (r −0.556, *p* < 0.001) in the first week of life. Linear regression analysis showed that zinc levels at 28 days of life were dependent on energy (β −0.650; *p* < 0.001) and protein (β −0.669; *p* < 0.001) intake received through PN in the first week of life, while no significant relations were found on the other confounding variables, described in the section named Statistical Analysis.

## 4. Discussion

Our findings suggest that early energy and protein intakes affect zinc levels in preterm newborns during the first month of life. The current nutritional practices for preterm neonates consist of administering high doses of protein and calories as soon as possible after birth. An increasing amount of evidence suggests that early aggressive nutrition may cause electrolyte imbalances during the first week after birth [19,20]. Those metabolic disturbances, triggered by early aggressive nutrition, define a specific condition of the premature infant described as “refeeding syndrome” [21]. Refeeding syndrome has been firstly reported as a commonly overlooked condition in cases of severe, acute electrolyte, fluid-balance, and metabolic abnormalities in chronically malnourished patients undergoing renutrition [21]. The increase in insulin production, once nutrition is started, has been described as the underlying mechanism of this condition [22]. More recently, the association of hypokalemia, hypocalcemia and hypophosphatemia in preterm infants has been correlated to the refeeding syndrome, linked not to a re-feed phenomenon but likely to a suboptimal provision of nutrients [7]. Bonsante et al. called that neonatal syndrome related to the interruption of the continuous nutritional placental flow, namely Placental Interrupted Feeding Syndrome of the preterm infants (PI-Feeding Syndrome). Our results confirmed the hypothesis that zinc metabolism is involved in PI-Feeding syndrome. Zinc consumption during anabolic processes might cause variations in its serum concentration in early life. Zinc is required for the activity of a number of proteins (i.e., enzymes, membrane proteins, gene-regulatory proteins, and hormonal receptors), it has a crucial role in maintaining quaternary structure stability of proteins and it participates in the regulation of gene expression, cell division and growth [3,23,24,25,26,27]. Consequently, during anabolic processes which result in higher protein synthesis, particularly after birth, the requirements of zinc increase.

In the absence of adequate supplementation, serum zinc concentrations decline during the first months of life [28,29,30,31]. Interestingly, recommended intakes have progressively increased during the last decade. The recommended enteral intake ranges between 0.8 to 3 mg/Kg/day [4]. Zinc requirements for term infants are estimated to be 0.8 mg/day, whereas preterm neonates may require up to 3 mg/kg per day to achieve adequate zinc retention [4]. Zinc content in human milk varies considerably (0.7 to 1.6 mg/L) and declines with time; while colostrum contains 8–12 mg/L, human milk at seven days of neonatal life contains 3–6 mg/L of zinc. These values rapidly decrease at 1–3 mg/L at 1 month of life [32,33,34,35,36]. Less variability regarding intravenous supplementation may be found in recommendations of different scientific societies and committees [33,37]. Given that only 60% of parenterally infused zinc is retained, different authors suggest parenteral supplementation with 350 mcg/kg per day [33,37]. Recent evidence supports the use of higher zinc doses to improve survival and reduce morbidity in VLBW neonates [38]. Since in the last trimester of pregnancy the fetus receives up to 1 mg/Kg/day of zinc from the mother, higher zinc supplementation seems to be necessary in post-natal life, especially for neonates born preterm. Zinc requirements in preterm neonates are quite similar to that of fetuses with similar postconceptional age. However, only a lower percentage of zinc administered in preterm neonates is retained. Thus, if zinc is provided according to current recommendations, the final balance of zinc may be negative. Our results support the hypothesis that an increased protein and energy intake should be associated with an increased zinc intake.

No previous research has investigated the relation between macronutrients intake in early life and zinc metabolism in preterm babies. Many clinical trials investigated the role of zinc in the treatment of acute diarrhea in association with others medications [39]. Zinc has been shown to be crucial for ensuring adequate growth. We observed increased growth velocity associated with lower levels of zinc in VLBW. The relation between growth and zinc levels has been reported in recent studies. Sjostrom et al. have demonstrated that there is no association between zinc intake and growth in the first month of life. However, in this retrospective study serum levels of zinc were not investigated and there was a lack of standardized methods in measuring growth parameters [40]. In a retrospective study on 59 preterm newborns, serum zinc levels were found to be inversely correlated with fetal growth parameters [41]. Despite no data on post-natal growth being reported, this study demonstrated a similar correlation between growth and zinc levels.

The main results of this study should be interpreted taking into account some limitations. Firstly, the present study has observational characteristics in the absences of a control group. Secondly, measurement of zinc in blood may be influenced by inappropriate sample handling, hemolysis of red blood cells in the serum, the time of the day when blood samples are collected (i.e., serum zinc is higher in morning samples than in evening samples). Additionally, serum zinc concentration decreases with a number of morbidities. However, multivariate analysis (that considered this aspect) confirmed the main result of the study. Finally, we evaluated a specific population of neonates, thus the conclusion may not be generalized to subjects with different GA, BW or clinical conditions.

## 5. Conclusions

In conclusion, our results suggest that zinc intake should be modulated in accordance with protein and energy parenteral intake administered in the first week of life. Preterm neonates may have higher parenteral requirements in zinc than previously recommended. If confirmed by further researches, this study suggests that the current PN guidelines need to reconsider zinc intake when increasing amino acids and energy intake during the first week of life.

## Figures and Tables

**Table 1:** Clinical characteristics of study population.

No. 103	
Antenatal steroids ^a^, No. (%)	58 (56.3)
Gestational age, weeks	31.5 ± 2.8
Birth weight, g	1634 ± 561
Length at birth, cm	44.5 ± 37.1
Head circumference at birth, cm	29.1 ± 2.8
Cesarean section, No. (%)	89 (86.4)
Male sex, No. (%)	57 (55.3)
Twins, No. (%)	29 (28.2)
1-min Apgar score	6 ± 2
5-min Apgar score	8 ± 1
pH at birth	7.2 ± 0.5
Base excess on cord blood, mmol/L	−5.0 ± 4.8
CRIB II score ^b^	3.0 ± 3.6
Exclusively breast milk at 28 days of life, No (%)	9 (8.7)
Morbidity overall, No (%)	28 (27.2)
NEC, No (%)	2 (1.9)
IVH, No (%)	5 (4.9)
PLV, No (%)	1 (1.0)
Sepsis proven by positive cultures, No (%)	5 (4.9)
ROP, No. (%)	15 (14.6)
BPD, No. (%)	6 (5.8)
Anaemia of prematurity, No. (%)	19 (18.4)
PDA, No (%)	16 (15.5)
Mortality, No (%)	2 (1.9)

Table legend: ^a^ Intramuscular steroids cycle in two doses of 12 mg over a 24-h period; ^b^ CRIB II: clinical risk index for babies, without temperature measures; NEC (Necrotizing Enterocolitis); IVH (Intraventricular Hemorrhage); PLV (Periventricular Leucomalacia); ROP (Retinopathy of Prematurity); PDA (Patent Ductus Arteriosus); BPD (Bronchopulmonary Dysplasia). Notes. Data were expressed as mean ± standard deviation, when not specified.

**Table 2:** Serum zinc levels, nutritional intake and growth according with body birth weight.

	Body Birth Weight < 1500 g	Body Birth Weight ≥ 1500 g	*p*
	(No. 41)	(No. 62)	
Zinc at birth, µg/dL	66.6 ± 10.3 *	66.6 ± 10.8 *	0.983
Zinc at 28 days of life, µg/dL	37.5 ± 13.5	47.2 ± 17.1	0.003
Decrease in Zinc **	0.4 ± 0.2	0.3 ± 0.3	0.005
Parenteral Nutrition Energy Intake, kcal/Kg/day	526.8 ± 103.8	151.1 ± 232.7	<0.001
Total Energy Intake, kcal/Kg/day	631.1 ± 135.3	533.4 ± 151.4	0.001
Parenteral Nutrition Protein intake, g/Kg/day	18.7 ± 3.9	4.9 ± 7.7	<0.001
Total Protein intake, g/Kg/day	21.2 ± 3.6	14.5 ± 5.0	<0.001
Growth Velocity #, g/Kg/day	11.5 ± 2.8	4.0 ± 7.3	< 0.001

Table legend: * vs Zinc at 28 days of life *p* < 0.05; ** (Zinc at birth—Zinc at 28 days of life)/Zinc at birth; # calculated at 36 weeks of PMA. Notes. Data were expressed as mean ± standard deviation.

**Table 3:** Decrease in serum ** zinc levels during the first 28 days of life according with energy and protein intake in the first week of life.

	Decrease in Zinc ** in Newborns Receiving High Intake in the First Week of Life	Decrease in Zinc ** in Newborns Receiving Low Intake in the First Week of Life	*p*
Calculated on Energy intake through Parenteral Nutrition	0.4 ± 0.2	0.2 ± 0.2	<0.001
Calculated on Total Energy intake	0.5 ± 0.2	0.2 ± 0.2	<0.001
Calculated on Protein intake through Parenteral Nutrition	0.5 ± 0.2	0.2 ± 0.2	<0.001
Calculated on Total Protein intake	0.5 ± 0.2	0.2 ± 0.2	<0.001

Table legend: ** (Zinc at birth—Zinc at 28 days of life)/Zinc at birth; Notes. Data were expressed as mean ± standard deviation.

## Supplementary Materials

The following are available online at https://www.mdpi.com/2072-6643/12/2/529/s1, Table S1: Parenteral Nutrition protocol.
