# Supplementary material for: Nutritional Intake Influences Zinc Levels in Preterm Newborns: An Observational Study
Source: Nutrients. 2020 Feb 19;12(2):529. doi: 10.3390/nu12020529 (PMC7071515; doi:10.3390/nu12020529)
Supplement: Supplementary file 1 [file nutrients-12-00529-s001.pdf]

Table S1. Parenteral Nutrition protocol.

		Birth weight < 1000 g	Birth weight ≥ 1000 g
**Energy** *(kcal/kg/day)*	Starting dose	55	60
	At day of life	0	0
	Target dose	120	110
	At day of life	7	7
**Proteins** *(g/kg/day)*	Starting dose	2.0	2.0
	At day of life	0	0
	Target dose	4.0	3.5
	At day of life	7	7
**Dextrose** *(g/kg/day)*	Starting dose	7.0	8.5
	At day of life	0	0
	Target dose	16.0	15.0
	At day of life	7	7
**Lipids** *(g/kg/day)*	Starting dose	2.0	2.0
	At day of life	0	0
	Target dose	4.0	3.5
	At day of life	7	7
